# Correction: Hassan et al. *Brassica juncea* L. (Mustard) *Extract Silver* NanoParticles and Knocking off Oxidative Stress, ProInflammatory Cytokine and Reverse DNA Genotoxicity. *Biomolecules* 2020, *10*, 1650

**DOI:** 10.3390/biom16040485

**Published:** 2026-03-24

**Authors:** Sohair Aly Hassan, Ali Mohamed El Hagrassi, Olfat Hammam, Abdelmohsen M. Soliman, Essam Ezzeldin, Wessam Magdi Aziz

**Affiliations:** 1 Therapeutic Chemistry Department, Pharmaceutical Industries Division, National Research Centre, 33 El Bohouth St., Dokki, Giza 12622, Egypt; solimanmohsen@yahoo.com (A.M.S.); wessamagdi@yahoo.com (W.M.A.); 2 Phytochemistry and Plant Systematics Department, Pharmaceutical Industries Division, National Research Centre, 33 El Bohouth St., Dokki, Giza 12622, Egypt; alielhagrasi@gmail.com; 3 Pathology Department, Theodor Bilharz Research Institute, P.O. Box 30, El Warraq, Giza Governorate 12411, Egypt; totoali1@hotmail.com; 4 Department of Pharmaceutical Chemistry, College of Pharmacy, King Saud University, P.O. Box 2457, Riyadh 11451, Saudi Arabia; esali@ksu.edu.sa

In the original publication [1], there was a mistake in Figure 5. There was a duplicated image in Figure 5F,H, where identical sections appear to be present. The corrected image appears below. The authors state that the scientific conclusions are unaffected. This correction was approved by the Academic Editor. The original publication has also been updated.

**Figure 5:**
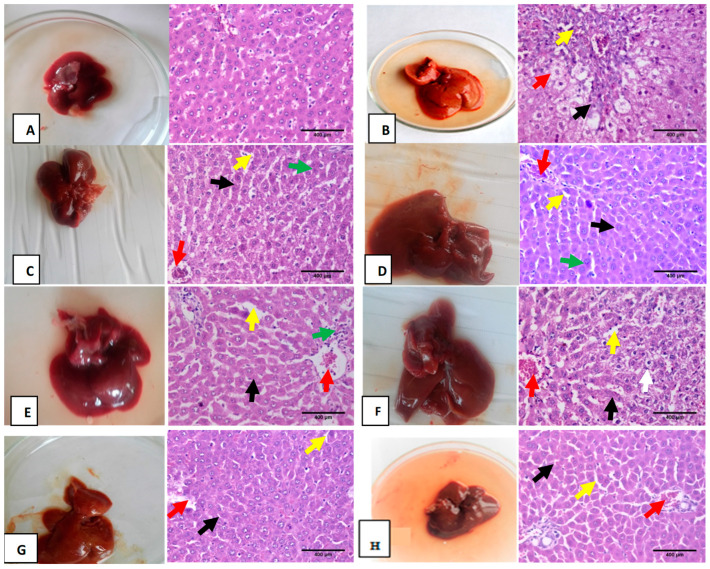
Photomicrograph of sections of hematoxylin and eosin (H and E, ×400) for liver tissues of different treated groups. (**A**) represents the sections from the normal control group with typical structure and morphology of the liver tissue; (**B**) TTA-intoxicated group showed severe abnormalities, partial distortion of lobular hepatic architecture (black arrow), with moderate hydropic degeneration of hepatocyte (red arrow) and thick fibrous tissue in portal tract (blue arrow); (**C**,**D**) liver sections treated with MS and nano MS showed hepatic tissue with almost normal intact hepatic lobular architecture and structure, hepatocytes arranged in thin plates (black arrow) and sinusoids (yellow arrow), central vein (red arrow), binucleated nuclei (green arrow); (**E**,**F**) liver sections treated orally with PMS nano PMS as a prophylactic group. They demonstrated tangible improvement as hepatic tissue was almost in normal intact hepatic lobular architecture and structure; hepatocytes arranged in thin plates (black arrow) with mild interlobular inflammatory infiltration (green arrow) and sinusoids (yellow arrow), congested central vein (red arrow), hepatocytes with moderate hydropic degeneration (white arrow); (**G**,**H**) liver sections treated with MS and MS nano groups showed improvement in hepatic tissues as it appeared in intact lobular hepatic architecture, hepatocyte with thin plates (black arrow), congested central vein (red arrow) and congested sinusoids (yellow arrow) ((**H**,**E**), ×400).
